# The application of Electrochemiluminescence Immunoassay (ECLIA) in the detection of HIV infectious markers in blood screening

**DOI:** 10.12669/pjms.41.10.12200

**Published:** 2025-10

**Authors:** Song He-Wei, Shen Xin-Tang, Hao Yi-Wei, Zuo Bin

**Affiliations:** 1 Song He-Wei, Yantai Central Blood Station, Yantai 264003, Shandong Province, China; 2 Shen Xin-Tang, Yantai Central Blood Station, Yantai 264003, Shandong Province, China; 3 Hao Yi-Wei, Yantai Central Blood Station, Yantai 264003, Shandong Province, China; 4 Zuo Bin, Yantai Central Blood Station, Yantai 264003, Shandong Province, China

**Keywords:** Blood screening, Confirmatory test, ECLIA, HIV, Window phase

## Abstract

**Objective:**

This study aimed to analyze ELISA, ECLIA, and Western blot (WB) confirmatory test results for HIV-reactive blood donors, compare ECLIA with fourth-generation ELISA reagents, assess the feasibility of using ECLIA for HIV screening, and provide statistical evidence supporting its adoption in blood collection and supply institutions.

**Methodology:**

The cross-sectional study was conducted in the Laboratory Department of Yantai Central Blood Station, Shandong, China, from January, 2021 to December, 2023. A total of 130 HIV screening-reactive specimens with ELISA signal-to-cut-off (S/CO) ratios between 0.3 and 0.7 were subjected to ECLIA. Specimens with ELISA S/CO≥1.0, HIV RNA reactivity, or ECLIA S/CO>1 were sent to the Centers for Disease Control (CDC) for WB confirmatory testing. The results were comprehensively analyzed and compared.

**Results:**

Among the 130 reactive specimens, 17 tested positive by ECLIA, while 113 were negative. Of the 510 HIV screen-negative specimens, five were positive by ECLIA, and 505 were negative. ECLIA demonstrated a sensitivity of 81.25%, specificity of 96.49%, positive predictive value of 76.47%, and negative predictive value of 97.35%. ECLIA showed excellent concordance with WB confirmatory results, evidenced by the highest area under the operating characteristic (ROC) curve (AUC). The sensitivities of reagent one, reagent two, and ECLIA were consistent at different cut-off values; however, ECLIA exhibited the highest specificity.

**Conclusion:**

ECLIA exhibits superior agreement with WB confirmatory tests, higher specificity, and positive predictive values compared to ELISA reagents, supporting its widespread use in blood screening.

## INTRODUCTION

Human immunodeficiency virus (HIV) infection not only severely compromises individual health but also poses a significant public health challenge. By the end of 2023, approximately 1.29 million people living with HIV/AIDS were reported in China, with about 110,500 newly diagnosed cases that year, and roughly 98.5% of these were attributed to sexual transmission.^1^Against this alarming backdrop, blood screening, as the final barrier against transfusion-transmitted HIV, is directly linked to the safety of recipients through its sensitivity and reliability. Although the overall HIV positivity rate remains low in some regions, strengthening laboratory testing capacity, adopting highly sensitive detection methods, and implementing appropriate screening strategies remain fundamental prerequisites for ensuring blood safety.^2^

However, mainstream HIV blood-screening methods, particularly the widely used enzyme-linked immunosorbent assay (ELISA), face a major challenge known as the “window period.”^3^ During this period, infected individuals may carry high viral loads and be contagious, but because antibodies have not yet developed or their levels remain too low, ELISA may yield false-negative results. Additionally, p24 antigen levels may decline before antibody titers peak, thereby posing a substantial risk of transfusion-transmitted infection.^4^ With the increasing number of HIV-infected individuals, the proportion of donors in the window period or with early infection may likewise rise, further amplifying the risk of missed detection.

Moreover, ELISA testing may produce false-positive results due to cross-reactivity or nonspecific binding, leading to the wastage of valuable blood resources, increased costs associated with repeat testing, and unnecessary psychological stress for donors. Variations in sensitivity and specificity also exist among ELISA kits from different manufacturers and across different generations.^5^ To mitigate these limitations, particularly to shorten the window period, blood establishments in China routinely use high-sensitivity fourth-generation ELISA kits in combination with nucleic acid testing (NAT). NAT, by directly detecting viral RNA, can reduce the window period to approximately 7-11 days post-infection, thereby significantly enhancing blood safety.^6^ However, NAT is associated with drawbacks such as high cost, operational complexity, and stringent laboratory infrastructure requirements.

The diagnostic performance of current testing methods can be affected by donors’ immune status, stage of infection, and the genetic diversity of HIV subtypes, potentially leading to false negatives or reduced detection sensitivity. Therefore, continuously optimizing testing strategies and exploring faster, more sensitive, and highly specific screening tools are crucial for adapting to changes in epidemiological patterns and the diverse blood donor population, as well as enhancing blood safety. Electrochemiluminescence immunoassay (ECLIA), an improved form of chemiluminescence immunoassay (CLIA), offers faster detection, higher throughput, and superior sensitivity and specificity. As a result, many blood collection organizations have begun to explore or gradually adopt high-performance CLIA/ECLIA systems to improve screening efficiency and safety.^7^-^10^ In China’s blood collection system, CLIA/ECLIA is still in the early stages. Currently, only a few CLIA kits have been approved by the National Medical Products Administration (NMPA), and their effectiveness in large-scale screening among Chinese blood donors requires further validation. This study aims to evaluate the potential of ECLIA as a supplementary method to the existing ELISA and NAT screening strategies. We compared ECLIA with current methods in terms of sensitivity, specificity, positive predictive value (PPV), negative predictive value (NPV), and window-period detection capability, providing evidence for optimizing HIV screening protocols and enhancing blood safety.

## METHODOLOGY

This was a retrospective cohort study conducted at the Laboratory Department of Yantai Central Blood Station, located in Yantai, Shandong Province, China. The study period was from January 2021 to December 2023. All data were obtained from routine HIV blood screening procedures performed at the institution. From January 1, 2021, to December 31, 2023; 179,916 blood donors who met the requirements of the *Health Examination Requirements for Blood Donors* (GB18467-2011; dated: June 4, 2025) in the Yantai area were included. The age of blood donors ranged from 18 to 60 years. Blood donors signed the *Informed Consent Form for Blood Donors* before donation.

### Reagents and Instruments:

ELISA: HIV-Ab/Ag detection reagent kit (hereinafter referred to as Reagent 1); HIV-Ab/Ag detection reagent kit (hereinafter referred to as Reagent 2); Swiss Hamilton STAR pipetting system, Swiss Hamilton FAME 24/20 enzyme immunoassay analyzer. NAT: Roche Cobas s201 nucleic acid testing system and supporting reagents (USA), Shanghai Haoyuan Chitas BSS1200 nucleic acid screening system and supporting reagents. ECLIA: Roche Cobas E411 electrochemiluminescence immunoassay analyzer (USA). All reagents were used within their validity period.

### Specimen Collection and Processing:

During the blood donation process, three specimens are collected using a satellite pouch method, with two 5mL tubes (containing EDTA-K2) for ELISA testing and one 5mL tube (containing EDTA-K2 anticoagulant with separating gel) for NAT testing. The NAT specimen should be centrifuged at 2000 g for 15 minutes within four hours after collection. All specimens were transported under cold chain conditions and stored in a refrigerator at 2~8°C. Testing should be completed within 72 hours. All tests were conducted strictly according to the corresponding reagent instructions and the latest version of the *Blood Station Technical Operation Procedures*.

### Testing Judgment Rules:

ELISA:If the S/CO of both reagent one and reagent two is<0.70, the specimen is considered qualified. If the S/CO of either reagent is ≥ 0.70, it is considered reactive. When the S/CO of either reagent is≥0.70, the original specimen and its blood bag sample should be retested using the corresponding reagent in duplicate wells. If the S/CO of any well in the retest is≥0.70, the result is considered unqualified. NAT: Specimens that were reactive in both ELISA reagents were not tested by NAT, while all other specimens were tested by NAT. Cobas s201 and Chitas BSS1200 were used for HBV/HCV/HIV combined nucleic acid testing (multiplex NAT, MP-NAT) with six and eight specimens pooled, respectively. If the pooled test result is non-reactive, the specimens were considered qualified. If the pooled test result is reactive, individual donor sample nucleic acid testing (ID-NAT) should be conducted. If the ID-NAT result is non-reactive, the specimen is considered qualified. If the ID-NAT result is reactive, the specimen is considered unqualified. ECLIA: Specimens with an initial screening S/CO≥0.3 in either reagent one or reagent two and ID-NAT reactive specimens were tested using ECLIA as a supplementary test. Specimens with an ECLIA test result<0.90 were considered non-reactive. Specimens with an ECLIA test result ≥0.90 should be retested. If the retest result is <0.90, the specimen is considered non-reactive. If the initial and retest ECLIA results are≥0.90, the specimen is considered repeatedly reactive. Confirmatory Testing: ELISA HIV Ag/Ab reactive specimens (S/CO≥1), ECLIA HIV Ag/Ab reactive specimens, and HIV RNA reactive specimens were sent to Yantai CDC for anti-HIV confirmatory testing using the Western Blot(WB) method.

### Statistical analysis:

Data is processed using Excel 2016 and statistical analysis is conducted using SPSS 23.0. To estimate the diagnostic sensitivity and specificity of the HIV screening method for blood donors, we used Buderer’s method and the sample size calculator provided by W.N. Arifin (https://wnarifin.github.io/ssc/sssnsp.html). Assuming a sensitivity of 80%, specificity of 95%, HIV prevalence of 0.05%, precision of ±10%, and a confidence level of 95%, the required total sample size to estimate sensitivity was 122,927, with a final sample size of 129,397 after accounting for a 5% dropout rate. In the actual study, a total of 179,916 blood donor records were included, which met the required total sample size. Differences in rates between groups are compared using the *Pearson chi-square* test and *Fisher’s* exact probability method, with *p*<0.05 considered statistically significant. Consistency of count data is assessed using the *Kappa* test, with *Kappa*≥0.75 indicating strong agreement, 0.75>*Kappa*≥0.4 indicating good agreement, and *Kappa*<0.4 indicating poor agreement. The S/CO values of reagent one, reagent two, and the ECLIA test values are used as statistical variables, and the Western blot (WB) confirmatory test results are used as state variables to draw a receiver operating characteristic curve (ROC). Analysis is conducted using the area under the curve (AUC), with *p*<0.05 considered statistically significant. Positive predictive value (PPV) and negative predictive value (NPV) were calculated using the following formulas: PPV=TP/(TP+FP), NPV=TN/(TN+FN),where TP=true positives, FP=false positives, TN=true negatives, and FN=false negatives.

## RESULTS

The test results of 179,916 blood samples are shown in Table-I: 135 cases were reactive with Reagent-1, Reagent-2, HIV RNA, and ECLIA, with a positive rate of 0.075% (135/179,916). The single-reagent reactivity rate of Reagent 1 (0.0072%) was higher than that of Reagent 2 (0.0534%), χ^2^=63.26, *p*<0.05.

**Table-I:** HIV blood screening results of 179,916 blood donors (n, %).

	ELISA HIV-/HIV RNA-(n,%)	ELISA HIV+/HIV RNA+(n,%)	ECLIA HIV+(n,%)
Reagent 1	179 885(99.983)	13*(0.0072)	0(0.000)
Reagent 2	179 802(99.937)	96*(0.0534)	3(13.636)
Reagent 1 + Reagent 2	179 789(99.929)	18**(0.0100)	14(63.636)
HIV RNA***	179 895(99.998)	3(0.0017)	/
ECLIA****	505(99.020)	0	5(22.727)

***Note:***

* indicates single-reagent reactivity;

** indicates dual-reagent reactivity;

*** excludes 18 cases of dual-reagent reactivity;

**** total specimen size is 510.

HIV blood screening, ECLIA, and WB confirmation test results showed (Table-II): The positive predictive value of ECLIA compared with Reagent 1, χ^2^=5.274, *p*<0.05, with a statistically significant difference. Consistency between ECLIA and WB confirmation tests: *Kappa*=0.757, indicating strong consistency between the two. ROC curves show (Fig.1, Table-III) that AUCs of Reagent-1, Reagent-2, and ECLIA reagent are 0.825, 0.829, and 0.853, respectively. All three reagents have good diagnostic capabilities, with ECLIA having the largest AUC.

**Table-II:** Comparison of consistency between four reagents and WB confirmation test results (n=130).

		Negative*	WB Confirmation Positive	PPV(%)	NPV (%)	*Kappa*	*²*	*p*
Reagent 1	-	96	3	41.94	96.97	0.467	33.108	0.000
	+	18	13					
Reagent 2	-	13	3	11.40	81.25	-0.020	0.702	0.417
	+	101	13					
NAT	-	109**	0	100	100	1.000	112.0	0.000
	+	0	3***					
ECLIA	-	110	3	76.47	97.35	0.757	74.601	0.000
	+	4	13					

***Note:*** "-" represents negative, "+" represents reactive.

* Negatives include donors with screening test results in the gray zone and those with negative/indeterminate WB confirmation test results.

** According to blood screening strategies, NAT testing is not performed for donors with dual-reagent ELISA reactivity in the initial screening, and the 109 NAT-negative donors do not include the 18 donors with dual-reagent ELISA reactivity in the initial screening.

*** Three donors were followed up by CDC, and the WB test was confirmed positivity.

**Fig-I:**
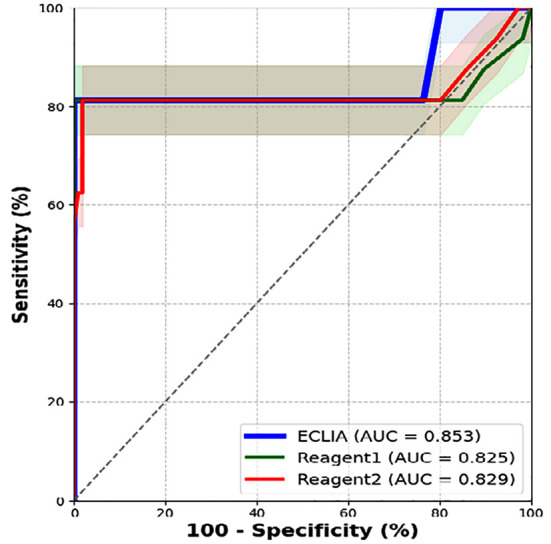
ROC curves for the test results of reagent 1, reagent 2, and ECLIA.

**Table-III:** Evaluation of the diagnostic effects of three reagents on HIV (n=122).

Reagent	Optimal Cut-off Value (S/CO)	Standard Error	AUC	95%CI	Sensitivity (%)	Specificity (%)	Maximum Youden Index
Reagent 1	13.22	0.091	0.825	0.646~1	81.3	100	0.813
Reagent 2	21	0.087	0.829	0.659~0.999	81.3	98.1	0.794
ECLIA	75.95	0.077	0.853	0.703~1	81.3	100	0.813

***Note:*** 8 cases with uncertain WB confirmation test results were not included in the ROC statistical analysis.

The sensitivity and specificity of the three reagents at different cut-off values are shown in Table-IV. When the S/CO value is 1.0 or the cut-off value is one, the specificities of reagent one, reagent two, and ECLIA are 72.6%, 61.3%, and 77.5%, respectively.

**Table-IV:** Sensitivity and specificity of the three reagents at different cut-off values (n=122).

Reagent	Optimal Cut-off Value			Kit-specified Cut-off Value			Laboratory-set Gray Zone Cut-off Value		
	Test Value	Sensitivity (%)	Specificity (%)	Test Value	Sensitivity (%)	Specificity (%)	Test Value	Sensitivity (%)	Specificity (%)
Reagent 1	13.22	81.3	100	1	81.3	72.6	0.7	81.3	60.4
Reagent 2	21	81.3	98.1	1	81.3	61.3	0.7	81.3	33.0
ECLIA	75.95	81.3	100	1	81.3	77.5	0.9	81.3	76.6

The current testing strategy combination is set as reagent 1, reagent 2+1 NAT, Strategy-1 combination is reagent 1+1 NAT, Strategy-2 combination is reagent 2+1 NAT, and Strategy-3 combination is ECLIA+1 NAT. The results show ( Table-V) : The number of confirmed positives for all four testing strategies is 16, with a confirmed positive rate of 0.0089%; The false positive rates of the current strategy, Strategy-1, Strategy-2, and Strategy-4 are 6.336%, 0.445%, 5.614%, and 0.222%, respectively; Strategy-3 has the highest positive predictive value (80.00%); Comparison of the positive predictive values between the current strategy and Strategy-3 shows χ^2^=22.346, *p*<0.05, with a statistically significant difference; comparison of the positive predictive values between Strategy-1 and Strategy-3 shows χ^2^=0.154, *p*>0.05, with no statistically significant difference; comparison of the positive predictive values between Strategy-2 and Strategy-3 shows χ^2^=19.393, *P*<0.05, with a statistically significant difference.

**Table-V:** Comparative Analysis of Blood Screening Strategies (n=640).

Testing Mode	Reactivity	Confirmed Positives	Confirmed Negative/ Indeterminate	False positive rate (%)	PPV(%)	NPV (%)
Current strategy	130	16	114	17.812	12.307	99.998
Strategy1	34	16	8	1.250	66.667	99.998
Strategy2	117	16	101	15.781	13.675	99.998
Strategy3	22	16	4	0.625	80.000	99.998

## DISCUSSIONS

In this study, we analyzed blood screening data from 179,916 unpaid blood donors in Yantai, comparing the performance of two ELISA reagents, one ECLIA reagent, and NAT, using WB as the confirmatory standard. Our findings demonstrated that ECLIA achieved comparable sensitivity to ELISA but outperformed it in specificity and positive predictive value (PPV), with superior detection capability in window period cases (Table-I, Table-II). ECLIA also showed the highest agreement with WB confirmation, indicating that its detection results were closest to the “gold standard.” ROC curve analysis revealed that ECLIA had a larger AUC than both ELISA reagents, with an optimal cut-off value of 75.95 (signal output) derived from ROC analysis, and it achieved the highest specificity. Comparison of screening strategies indicated that Strategy 3 had the highest PPV (80.00%) and the lowest false-positive rate (Table-V). These results suggest that incorporating ECLIA into the current screening system can maintain sensitivity while significantly improving specificity and PPV, reducing blood wastage due to false positives, and offering strong potential for use in high-throughput laboratories.

Compared with other regions in China, the confirmed HIV positivity rate among unpaid blood donors in Yantai (0.0089%) was notably lower than that reported in Shenzhen between 2013 and 2018 (0.027%-0.073%)^11^ and Guangzhou between 2003 and 2022 (0.0189%),^12^ and was comparable to that observed in Nanchang from 2021 to 2024 (0.0091%).^13^ This difference may be attributable to stricter donor selection criteria and more standardized pre-donation counseling in medium and smaller cities such as Yantai, which reduce the likelihood of high-risk individuals entering the blood donation system. Our findings are consistent with those from Jinan, where the Wantai CMIA reagent achieved a 100% detection rate for confirmed HIV-positive samples and 97.03% for early HIV RNA-positive cases, significantly outperforming ELISA (97.62%).^14^ Similarly, Wang et al. (2024) evaluated the MAGLUMI HIV Ab/Ag Combi assay in over 5,000 blood donors and reported a specificity of 99.85% and a sensitivity of 100%.^15^ Multicenter studies in China have also confirmed the stable and superior performance of ECLIA in donor screening; in a study involving 14 blood centers (n=1,029), ECLIA achieved 100% sensitivity, 99.0% specificity, a PPV of 93.8%, and a Kappa coefficient of 0.963, significantly outperforming ELISA.^16^ Beyond domestic findings, evidence from multiple international studies further supports the advantages of ECLIA. International evidence also supports the superior performance of ECLIA. In Europe, South Africa, and the Netherlands, the Elecsys HCV Duo assay achieved 99.6% sensitivity and 99.94% specificity in 20,634 samples,^17^ with identical results reported in Switzerland and Germany.^18^ In India, the Vitros ECi ECLIA outperformed ELISA and rapid tests in multi-marker screening for HBV, HCV, and HIV.^19^ In France, blood center evaluations confirmed the suitability of Elecsys HIV Duo and various HCV, HBV, and syphilis assays for first-time donor screening.^20^,^21^ In the United States, the FDA has approved the Roche Elecsys HIV Duo ECLIA for screening whole-blood, component, and plasma donors, and institutions such as the Mayo Clinic have incorporated it into routine HIV-1/2 screening.^22^ In Australia, the Lifeblood system uses combined NAT+ECLIA screening, reducing the residual risk of transfusion-transmitted HIV to approximately one per 31.7 million units.^23^ In Japan, ECLIA achieved 100% sensitivity and 99.8% specificity for HTLV screening, providing a technical reference for HIV ECLIA implementation.^24^ Collectively, domestic and international evidence demonstrates that ECLIA offers excellent sensitivity, specificity, and stability, underscoring its strong potential for widespread adoption in blood donor screening programs.

Since the nationwide implementation of NAT in China, the residual risk of transfusion-transmitted HIV has been significantly reduced, though it cannot be entirely eliminated.^25^ Therefore, ensuring clinical blood safety while continuously exploring more reliable screening methods remains a critical task for blood collection and supply institutions. The Technical Operating Procedures for Blood Stations (2019 Edition), issued under the guidance of the NMPA, explicitly state that CLIA can be used for serological testing.^26^,^27^ CLIA is characterized by high sensitivity, high specificity, good reproducibility, and full automation,^28^ in addition to low interference, stable conjugates, and reduced reagent consumption,^29^ making it well-suited for high-throughput laboratories. ECLIA, a subclass of CLIA, uses closed reagent systems and does not require separate sample-loading devices, minimizing manual errors and enabling a rapid detection time of approximately 20 minutes—considerably faster than ELISA.^30^

Our findings demonstrate that ECLIA offers clear advantages in detecting window period samples and reducing false-positive rates, particularly when used in combination with NAT or fourth-generation ELISA, effectively shortening the diagnostic blind period during early infection and enhancing overall blood safety.^31^ Furthermore, the higher specificity and PPV of ECLIA help reduce repeat testing and the unnecessary discarding of safe blood units, conserving resources and alleviating psychological stress on donors.^32^ In real-world applications, integrating ECLIA into high-throughput laboratories not only shortens detection times but also improves early infection detection capacity, thereby providing a robust technical foundation for developing a risk-based, evidence-driven national HIV screening strategy.

### Strength of study:

The main innovations of this study are reflected in three aspects: First, it provides the first systematic evaluation of ECLIA’s clinical application in the Yantai region. Second, it establishes an optimized screening strategy based on large-scale sample data. Third, it demonstrates ECLIA’s unique advantages in balancing detection sensitivity and blood resource utilization.

### Limitations:

This study has several limitations. The single-center design may affect the generalizability of the results. Additionally, the number of confirmed positive samples is limited (n=16), and some window-phase samples may have escaped detection. Future research should focus on: (1) multicenter studies with larger sample sizes; (2) systematic cost-effectiveness analyses; and (3) development of region-specific screening protocols. These findings provide robust evidence to support ECLIA implementation in China’s blood bank system. Particularly in low-prevalence regions like Yantai, ECLIA-based screening offers an effective approach to maintain detection sensitivity while optimizing resource utilization, which holds significant practical value for blood safety management.

## CONCLUSIONS

This study demonstrates that ECLIA achieves accuracy comparable to WB confirmatory testing, with higher specificity and PPV than ELISA and a lower false-positive rate. When combined with NAT, it can maintain sensitivity while improving specificity and reducing blood wastage.^33^ Given its superior performance and feasibility, ECLIA is expected to see broader adoption in national blood screening and to support risk-based combined testing strategies for optimizing HIV screening protocols.

### Authors’ Contributions:

**SH:** Conceived the study, designed the research protocol, conducted experiments, performed data analysis, and drafted the manuscript. He is responsible and accountable for the accuracy and integrity of the work.

**SX:** Conducted laboratory procedures and assisted in statistical analysis.

**HY:** Performed statistical analysis and contributed to data processing.

**ZB:** Supervised the study academically and critically revised the manuscript.

All authors have read and approved the final version of the manuscript.
